# Imaging Protein Misfolding in the Brain Using β-Sheet Ligands

**DOI:** 10.3389/fnins.2018.00585

**Published:** 2018-08-21

**Authors:** Ryuichi Harada, Nobuyuki Okamura, Shozo Furumoto, Kazuhiko Yanai

**Affiliations:** ^1^Department of Pharmacology, Tohoku University Graduate School of Medicine, Sendai, Japan; ^2^Division of Pharmacology, Tohoku Medical and Pharmaceutical University, Sendai, Japan; ^3^Cyclotron and Radioisotope Center, Tohoku University, Sendai, Japan

**Keywords:** proteinopathies, protein aggregates, β-sheet ligands, PET, tau

## Abstract

Neurodegenerative diseases characterized by pathological protein accumulation in cells are termed “proteinopathies.” Although various protein aggregates share cross-β-sheet structures, actual conformations vary among each type of protein deposit. Recent progress in the development of radiotracers for positron emission tomography (PET) has enabled the visualization of protein aggregates in living brains. Amyloid PET tracers have been developed, and are widely used for the diagnosis of Alzheimer’s disease and non-invasive assessment of amyloid burden in clinical trials of anti-dementia drugs. Furthermore, several tau PET tracers have been successfully developed and used in the clinical studies. However, recent studies have identified the presence of off-target binding of radiotracers in areas of tau deposition, suggesting that concomitant neuroinflammatory changes might affect tracer binding. In contrast to amyloid and tau PET, there are no established tracers for imaging Lewy bodies in the human brain. In this review, we describe lessons learned from the development of PET tracers and discuss the future direction of tracer development for protein misfolding diseases.

## Introduction

Proteinopathies are neurodegenerative diseases characterized by pathological accumulation of amyloid-β (Aβ), tau, α-synuclein, and TDP-43 in the cells. Tauopathies, characterized by tau protein accumulation, include Alzheimer’s disease (AD), some variants of frontotemporal lobar degeneration (FTLD), frontotemporal dementia with parkinsonism linked to chromosome-17 (FTLD-17), corticobasal degeneration (CBD), progressive supranuclear palsy (PSP), argyrophilic grain disease (AGD), and chronic traumatic encephalopathy (CTE). AD is a mixed proteinopathy characterized by abnormal accumulation of extracellular Aβ and intracellular tau protein. α-Synucleinopathies, characterized by pathological accumulation of α-synuclein, include Parkinson’s disease (PD), Lewy bodies disease (LBD), and multiple system atrophy (MSA). TDP-43 proteinopathies include some variants of FTLD and amyotrophic lateral sclerosis (ALS). Genetic, pathological, and biochemical evidence strongly suggests that protein accumulation causes neurodegenerative disease, although the precise etiology and mechanisms underlying these processes remain unknown. Historically, these neuropathological lesions could only be identified by histopathological examination at autopsy. Neuropathologists described the distributions of these lesions in many patients and proposed a classification of their regional distribution (Thal et al., 2002; Braak et al., 2006a; Hyman et al., 2012). Braak staging, which is based on cross-sectional autopsy studies, represents the most commonly used classification system for Aβ, tau, α-synuclein, and TDP-43, (Braak and Braak, 1991; Braak et al., 2003; Brettschneider et al., 2013). Importantly, the amount and spatial distribution of protein depositions is highly correlated with the severity of disease (Bierer et al., 1995; Braak et al., 2006b). Non-invasive detection of abnormal protein deposits would therefore be useful for accurate diagnosis, disease monitoring, and evaluation of the efficacy of anti-dementia treatments.

Positron emission tomography (PET) using specific radiotracers provides regional and pathophysiological information in a non-invasive manner in living human subjects. Although PET imaging is more expensive than examining body fluid biomarkers, such as blood and cerebrospinal fluid (CSF), it is advantageous because it enables the spatial assessment of brain lesions as well as accurate, reliable, and reproducible quantitative measurements. Following the successful imaging of amyloid with Pittsburgh compound B (PiB; Klunk et al., 2004), fluorinated alternatives have been developed in clinical studies and approved by the Food and Drug Administration (FDA) and European Medicines Agency (EMA; Mathis et al., 2017). These tracers are thioflavin and stilbene derivatives, which bind the cross-β-sheet structures of aggregated proteins. Many β-sheet binding compounds have been reported through the development of amyloid PET tracers (Furumoto et al., 2007). Several putative tau PET tracers have also been developed and evaluated in humans. In a clinical trial, these tracers demonstrated elevated retention in brain regions susceptible to tau deposition (Villemagne et al., 2015). However, tau pathology is more complicated than Aβ pathology, owing to its heterogeneous histopathology possibly resulting from isoform composition and ultrastructural conformation (for in depth review see Villemagne et al., 2012; Harada et al., 2016c). Furthermore, first generation tau tracers such as ^11^C-PBB3, ^18^F-AV1451 (also known as flortaucipir; T807), and ^18^F-THK5351 result in off-target binding (Okamura and Yanai, 2017). Recently, ^18^F-THK5351 was reported to bind with high affinity to monoamine oxidase-B (MAO-B), contributing to a high level of *in vivo* PET signal (Harada et al., 2017; Ng et al., 2017). In this review, we describe our experiences and lessons learned from the development of tau PET tracers, and discuss the future of radiotracer development for other proteinopathies.

## Characteristics of Protein Aggregates

**FIGURE 1:**
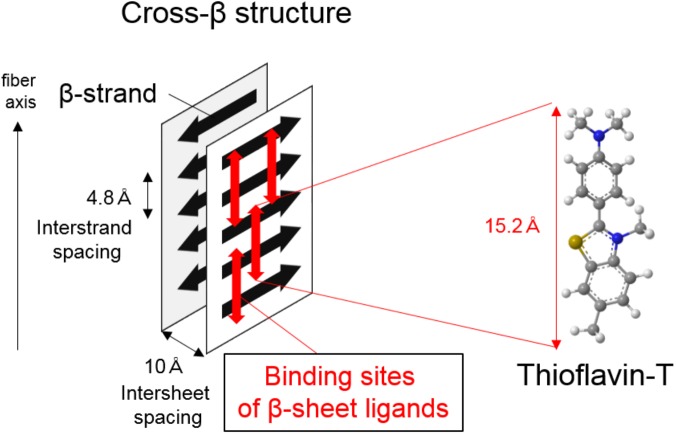
Illustration of cross-β structure in protein aggregates and proposed binding mechanism of β-sheet ligands such as thioflavin-T.

Amyloid-β, tau, and α-synuclein are highly insoluble when accumulated in the diseased brain. They comprise amyloid, a filamentous protein which has a cross-β-sheet structure in which individual β-strands run perpendicular to the fiber axis (Serpell et al., 2000; **Figure 1**). In most cases, these protein aggregates can be stained with histological dyes, such as Congo red and thioflavin-S, which bind the cross-β-sheet structures. Tau aggregates are hyperphosphorylated, and form twisted filaments called paired helical filaments (PHFs), which have a diameter of 8–20 nm and a stereotypical periodicity of 80 nm as observed by electron microscopy (EM). X-ray diffraction of amyloid plaques and PHFs from AD brains reveals unique characteristics, including a sharp reflection at 4.76-Å spacing and a diffuse reflection at about 10.6-Å spacing (Kirschner et al., 1986). However, there are various isoforms of tau in the human brain, leading to diverse ultrastructures, spatial distributions, and clinical phenotypes in tauopathies. Tau deposits in neurodegenerative diseases contain different isoform compositions: 3 repeat (3R) and 4 repeat (4R) tau in AD, predominantly 4R tau in CBD and PSP, and 3R tau in Pick’s disease. This leads to different affected cell types (e.g., neurons, astrocytes, oligodendrocytes) and histopathologies (e.g., neurofibrillary tangles and neuropil threads in AD, astrocytic plaques in CBD, tufted astrocytes in PSP, and Pick’s bodies in Pick’s disease). Ultrastructural and biochemical analyses reveal that the detailed composition and structure of tau deposits differ between CBD and PSP, despite both of these diseases being 4R tauopathies (Arai et al., 2004; Taniguchi-Watanabe et al., 2016). Because protein aggregates are highly insoluble, it is difficult to resolve the detailed structure of native aggregates in human brain tissue using conventional techniques such as X-ray crystallography and nuclear magnetic resonance (NMR). Thus, it is difficult to design rationally selective PET tracers. However, a recent study using a novel single particle cryo-EM approach revealed the detailed structure of native tau filaments from an AD brain (Fitzpatrick et al., 2017). The structure of the β-helix or the protofilament interface of PHF-tau may prove to be binding sites for selective tau PET ligands, owing to their specificities. Although the binding sites of tau PET ligands are complicated (Cai et al., 2016), this breakthrough may help in understanding ligand-protein interactions, and accelerate the development of new rationally designed PET tracers. Indeed, a Nobel Prize in chemistry was awarded for cryo-EM in 2017 (Bertozzi, 2017), supporting the potential of this technique to further the development of new tracers.

## Properties Required for Pet Tracers

Protein aggregates consisting of Aβ, tau, and α-synuclein predominantly form cross-β sheet structures, laminated assemblies of secondary structure β-sheets. Thus, β-sheet ligands such as thioflavin-T could be useful in the development of PET tracers for imaging protein aggregates. One of the most well-described β-sheet ligands is thioflavin-T, which is considered to bind with long axis of amyloid fibrils, having cross β-sheet structures in which individual β-strands run perpendicular to the fiber axis (**Figure 1**; Krebs et al., 2005). As described previously (Villemagne et al., 2012; Okamura et al., 2014b), useful neuroimaging radiotracers must possess the following chemical and pharmacological properties (summarized in **Table 1**): brain–blood barrier (BBB) permeability, ability to undergo rapid clearance from normal brain tissue and blood, and ability to bind with high affinity and specificity to targets.

**Table 1:** Properties required for PET tracers.

Properties	Requirements
High blood–brain barrier permeability	>4%ID/g at 2 min post injection in normal mice
Rapid clearance	2–30 min brain uptake ratio in mice > 10
High binding affinity	Kd or Ki < 20 nM for target(s)
Off-target binding	None
Reversible binding to target	Reversible binding to target(s)
Stability	No radiolabeled metabolites in the brain

Positron emission tomography radiotracers are often radiolabeled with ^11^C or ^18^F. In general, ^11^C-labeled tracers are easier to design, as ^11^C can be introduced into a methyl group. However, ^11^C-labeled tracers must be radiolabeled in each PET center, owing to the short half-life of ^11^C (*t*_1/2_ = 20 min). On the other hand, ^18^F has a longer half-life (*t*_1/2_ = 110 min), allowing for easier commercial application.

Binding affinity of a ligand for its target is a critical factor in *in vivo* imaging. Binding sites (*B*_max_) and binding affinity (dissociation constant; *K*_d,_) can be predicted using *in vitro* saturation binding assays in homogenized tissue. At steady state, (*B*_max_/*K*_d_) is used to calculate the binding potential (BP), and can be estimated using PET data. The rate constants for dissociation (*k*_off_; min^−1^) and association (*k*_on_; nM^−1^min^−1^) can also be predicted as an index of binding affinity and kinetics. The dissociation constant (*K*_d_) is calculated as the ratio (*k*_off_/*k*_on_). High-affinity ligands are characterized by low *k*_off_ and high *k*_on_, yielding a low *K*_d_. These parameters serve as a good index of the binding properties of PET radiotracers. The most successful neuroimaging radiotracers for imaging amyloid possess a high binding affinity with *K*_d_ values of less than 10 nM (Mathis et al., 2003; Choi et al., 2009; Ni et al., 2013). However, reports of these parameters in the literature should be carefully interpreted as they largely depend on assay conditions. Dissociation constants (*K*_d_) can also be assessed using *in vitro* autoradiography of human brain sections (Zhang et al., 2012; Maruyama et al., 2013; Xia et al., 2013). Binding inhibition constant (*K*_i_) is alternatively estimated if test compounds are structurally related to one radioligand and could be a good indicator of binding affinity for the efficient screening of a number of non-radiolabeled candidate compounds (Furumoto et al., 2007). Recombinant proteins are widely used in binding assays for screening protein-ligand interactions (Mathis et al., 2003; Fodero-Tavoletti et al., 2007; Thompson et al., 2009). However, assays using native protein aggregates derived from human brain tissue represent a more reliable method for measuring the binding affinities of ligands.

Several misfolded proteins share cross β-sheet structures and are present in the neocortex in neurodegenerative diseases (e.g., Aβ and tau in AD, α-synuclein in LBD). In case of AD, there are far fewer tau aggregates than Aβ aggregates in the cortex (5–20 times fewer; Villemagne et al., 2012). Therefore, tau PET tracers require 20–50 times higher selectivity than Aβ, as estimated by simulation studies (Schafer et al., 2012). Further, the density of binding sites in α-synuclein and TDP-43 aggregates is much lower than that in tau and Aβ aggregates. *In vitro* autoradiography of human brain tissue is a reliable method to assess the binding selectivity of radioligands if the assays are performed at low nanomolar ligand concentrations, such as those achieved in brain tissue during a PET scan. *In vitro* autoradiography can also identify non-specific binding of radiotracers. However, a change in the assay conditions, such as washing and fixation procedures, can affect *in vitro* autoradiography results. For example, ethanol is commonly used in the differentiation process to mask non-specific and off-target binding. However, this treatment is not representative of physiological conditions and may damage the native structures of proteins. Fixation of brain tissue also affects autoradiography results. Pathologists generally use formalin-fixed paraffin embedded tissue for diagnostic procedures; however, the process of fixation may affect the native conformation of target and non-target proteins.

In order to allow for BBB penetration via passive diffusion, PET tracers should be small molecules (<450 Da). Useful PET radiotracers show initial brain uptake of more than 5% of an intravenously injected dose. Initial brain uptake depends not only on BBB penetration, but also blood flow, plasma radiotracer concentration, and free fraction of the radiotracer in the plasma and brain. Lipophilicity is a critical property that determines brain uptake. Ideally, a radiotracer should be moderately lipophilic, with an octanol-water partition coefficient (log*P* value) in the range of 0.9–2.5. Rapid clearance from normal tissues without non-specific binding is also desirable, as slower pharmacokinetics prolong the time it takes to reach steady state in a PET scan. In preclinical studies, the ratio of brain uptake 2–30 min after intravenous injection of tracers is a useful index of the clearance of tracers from normal tissues. Furthermore, radiotracers for neuroimaging should be stable and metabolized peripherally. Radiolabeled metabolites should be polar, and exhibit no brain entry or interaction with target proteins. In ^18^F-labeled tracers, defluorination can cause the accumulation of ^18^F in the bone, which could interfere with the visual assessment and quantification of radiotracer binding.

## Screening of β-Sheet Ligands

As previously mentioned, small organic compounds that possess a high affinity for β-sheet structures are potential candidates for PET tracers in the imaging of proteinopathies. Planar compounds potentially bind to the cross β-sheet structure of protein aggregates. Congo red, thioflavin-S, and thioflavin-T are histological dyes that have traditionally been used for the visualization of amyloid lesions. Therefore, much effort has been made to optimize derivatives of these in the development of amyloid PET tracers. The first successful radiotracer for Aβ is ^11^C-PiB, a thioflavin-T derivative (Mathis et al., 2012). ^18^F-labeled amyloid PET tracers such as Florbetapir (Amyvid^TM^), Flutemetamol (Vizamyl^TM^), and Florbetaben (NeuroCeq^TM^) have also been developed and approved by the FDA and EMA. Identifying lead compounds for imaging proteinopathies other than Aβ has proven challenging, as most β-sheet binding ligands have a high affinity for Aβ fibrils. Although protein aggregates share a predominantly cross-β-sheet structure, they have distinct conformations. In fact, several fluorescent compounds display different emission spectra when bound to Aβ plaques and tau pathology in AD brain sections, suggesting that these compounds would interact with Aβ and tau aggregates differently (Harada et al., 2014). Fluorescence staining is useful for evaluating binding ability for initial screening processes. However, this method is very low throughput, and requires postmortem human brain samples. Previously, synthetic fibrils generated from recombinant proteins such as tau protein were used for the binding evaluation, but they cannot be expected to fully recapitulate the binding to native pathology. In fact, we have experienced the discrepancy of the binding data between synthetic fibrils and tissues containing native conformation of protein aggregates (Zhang et al., 2012; Okamura et al., 2013). Therefore, researchers have attempted to develop efficient and reliable high-throughput screening assays to identify lead compounds for imaging proteinopathies such as tau, α-synuclein, and TDP-43 aggregates. For example, tissue-based high throughput screening utilized with mass spectrometry imaging have been proposed (Yoshimi et al., 2015), but this field seems to be a big challenge.

## Development of Tau Pet Tracers

**FIGURE 2:**
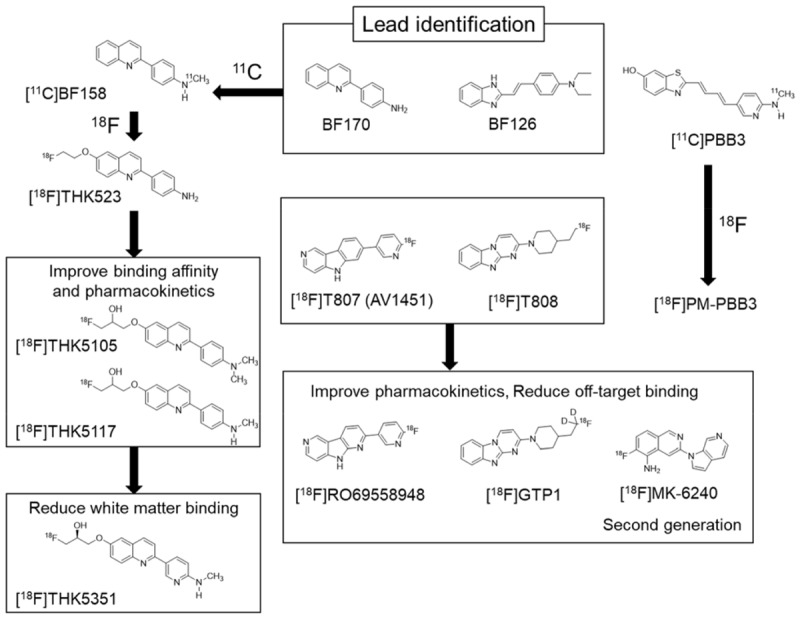
Chemical structures and flowchart of the development of tau PET tracers.

Compared with the development of amyloid PET tracers, there was no lead compound available in the development of selective PET tracer for tau or α-synuclein. In the case of tau, Okamura et al. (2005) identified quinoline and styryl-benzimidazole derivatives that clearly stained neurofibrillary tangles, neuropil threads, and dystrophic neurites in AD brain sections. After successful identification of these derivatives, a quinoline derivative ^11^C-BF158 was synthesized and evaluated in human brain sections. *In vitro* autoradiography of ^11^C-BF158 revealed preferential binding in tau-rich regions over Aβ-rich regions (Okamura et al., 2005). In addition, ^11^C-BF158 showed high BBB permeability in mice, suggesting that quinoline derivatives could be good candidates for tau PET tracers (**Figure 2**). Additionally, we synthesized the ^18^F-quinoline derivative ^18^F-THK523. Small animal PET studies have demonstrated higher tracer retention in rTg4510 tau transgenic mice compared to littermate controls (Fodero-Tavoletti et al., 2011). Direct comparison of ^18^F-THK523 with ^11^C-PiB, ^18^F-BF227, and ^18^F-FDDNP in AD brain sections revealed different binding distributions of ^18^F-THK523, which co-localized with tau immunostaining and Gallyas Braak silver staining (Harada et al., 2013). A first-in-human study of ^18^F-THK523 demonstrated significant tracer retention in the temporal parietal, orbitofrontal lobe, and hippocampus of AD patients, and different tracer retention compared to ^11^C-PiB in the same subjects (Villemagne et al., 2014). However, ^18^F-THK523 retention was significantly lower in gray matter than in white matter, suggesting insufficient binding affinity of this tracer to PHF-tau in the AD brain.

We further optimized the quinoline derivatives (Tago et al., 2014; Furumoto et al., 2017) and developed ^18^F-THK5105 and ^18^F-THK5117, which possess higher affinity for tau fibrils and better pharmacokinetics than ^18^F-THK523 (Okamura et al., 2013; Lemoine et al., 2015). These tracers were evaluated in human studies (Okamura et al., 2014a; Harada et al., 2015). In these clinical studies, radiotracer retention was elevated in the inferior temporal lobe of patients with AD where tau pathology was frequently observed at autopsy. ^18^F-THK5105 retention in the neocortex was significantly correlated with disease severity of AD patients (Okamura et al., 2014a). The spatial pattern of ^18^F-THK5117 retention in AD was consistent with that of ^18^F-THK5105, and different from that of ^11^C-PiB. However, both ^18^F-THK5105 and ^18^F-THK5117 were retained in white matter. As observed in amyloid PET tracers, β-sheet ligands may bind to white matter in a non-specific manner, owing to the presence of β-sheet-rich myelin basic protein (Stankoff et al., 2011). Lipophilicity of compounds can strongly influence white matter binding. For example, increasing hydrophilicity via the introduction of nitrogen to a benzene ring can reduce non-specific binding of the tracer to white matter, as observed using ^11^C-AZD2184, a pyridine derivative of ^11^C-PiB (Johnson et al., 2009; Nyberg et al., 2009).

To reduce non-specific tracer retention in white matter, we additionally developed ^18^F-THK5351, a pyridine derivative of ^18^F-THK5117 (Harada et al., 2016b; Tago et al., 2016b). Indeed, ^18^F-THK5351 is more hydrophilic than ^18^F-THK5117 (Log *P* = 1.5 vs 2.3). As expected, ^18^F-THK5351 showed less white matter binding and improved pharmacokinetics. A fluoropropanol side chain of THK5117 contains a chiral center. ^18^F-THK5117 is a racemic compound with equal amounts of *S*- and *R*-enantiomers. In general, each enantiomer exhibits different biological properties including binding affinity, pharmacokinetics, and metabolism. In our analysis, *S*-enantiomers of THK compounds were superior to *R*-enantiomers with respect to pharmacokinetics, tolerance to defluorination, and binding affinity to human brain homogenates (Tago et al., 2016a,b). In ^18^F-THK5351 PET study, we observed prominent tracer retention in the inferior temporal cortex of AD patients. ^18^F-THK5351 successfully reduced non-specific retention in white matter and exhibited superior pharmacokinetics than ^18^F-THK5117 (Harada et al., 2016b; Betthauser et al., 2017). The major radiolabeled metabolite of this tracer was a sulfate conjugate, which appears to be generated outside of (and does not enter into) the brain (Harada et al., 2016a).

In clinical studies of AD patients, regional tracer retention correlates well with glucose hypometabolism. and mirrors the clinical and neuroanatomical variability in AD variants (Chiotis et al., 2016; Saint-Aubert et al., 2016; Kang et al., 2017) as observed in ^18^F-AV1451 PET studies (Ossenkoppele et al., 2016). Furthermore, prominent tracer retention is observed in sites susceptible to tau deposition in non-AD tauopathies such as CBD and PSP (Chiotis et al., 2016; Kikuchi et al., 2016; Ishiki et al., 2017). However, ^18^F-THK5351 also accumulates in the temporal cortex in semantic variant primary progressive aphasia (svPPA), although notably, more than 90% of svPPA cases are FTLD-TDP type C pathology (Spinelli et al., 2017).

Since tau PET ligands recognize cross-β sheet structure of PHF-tau, they could detect extracellular ghost tangles as well as intracellular neurofibrillary tangles. However, they are not sensitive to non-argyrophilic pretangles that contain AT8-immunoreactive soluble tau (Braak et al., 1994; Sun et al., 2002). Tau immunohistochemistry and Gallyas Braak silver staining detect neurofibrillary pathology in layers II, III, and V of the neocortex in AD patients (Braak et al., 2006a). Interestingly, ^3^H-THK5351 binds preferentially to neurofibrillary tangles in the pyramidal cell layer (V) compared to the granule cell layers (II/III), although the granule cell layers also contain neurofibrillary tangles. Similar binding characteristics were observed for ^18^F-AV1451 (Lowe et al., 2016). These observations suggest variation in the structural conformation and maturity of neurofibrillary tangles in AD brains. As described by Lowe et al. (2016), this has very important implications on the sensitivity of tau PET imaging at different stages; tau PET ligands may detect more mature tangles than anticipated.

^18^F-THK5351 satisfies the requirements for neuroimaging PET tracers. However, ^18^F-THK5351 exhibits off-target binding in the basal ganglia and thalamus. Recent blocking studies have identified MAO-B as an off-target binding substrate of ^18^F-THK5351. In a human study, a single oral dose (10 mg) of selegiline, an MAO-B inhibitor, reduced ^18^F-THK5351 signal in the basal ganglia as well as neocortex (Ng et al., 2017). In addition, an autopsy imaging study of ^18^F-THK5351 in an AD patient demonstrated significant correlation of *in vivo* ^18^F-THK5351 signals with MAO-B levels (Harada et al., 2017). This indicates that ^18^F-THK5351 is not a selective PET tracer for tau. In our previous studies, THK5351-MAO-B binding was overlooked in human brain sections because the formaldehyde-fixed sections had been used in autoradiography analyses (Okamura et al., 2013; Harada et al., 2015, 2016b). Fixation of tissues with formalin, paraformaldehyde, or alcohol denatures native protein structures and deactivates native enzyme activity, while protein deposits such as neurofibrillary tangles and amyloid plaques are relatively stable. In fact, ^3^H-THK5351 binding in the putamen disappears after fixation with 4% paraformaldehyde, while laminar THK5351 binding in the neocortex remains detectable even after fixation. Therefore, selectivity of radiotracers should be assessed in unfixed frozen tissue. A receptor panel screen (i.e., ligand binding assay) has also been utilized to identify off-target binding of tracer candidates. However, functional assays are commonly used to assess a compound’s binding ability to a particular enzyme, and the panel screen was originally designed for the development of therapeutic drugs. In our experience, functional assays are less sensitive in the evaluation of the binding ability of a compound to an enzyme compared to ligand-binding assays.

^18^F-AV1451 (also known as flortaucipir, T807) was developed as tau PET tracer (**Figure 2**). This tracer showed high binding affinity and specificity to tau deposits in AD brain sections. ^18^F-AV1451 has been clinically evaluated in many PET centers (Villemagne et al., 2015) revealing a robust difference between AD patients and normal elderly controls. The spatial distribution of ^18^F-AV1451 followed the known distribution of tau aggregates in the neocortex, glucose hypometabolism in FDG-PET, and cortical atrophy in MRI (Schwarz et al., 2016; Hanseeuw et al., 2017; Xia et al., 2017). ^18^F-AV1451 PET studies have also demonstrated a distinct pattern of tracer binding in non-AD tauopathies such as PSP, CBD, and microtubule associated tau (MAPT) mutation carriers (Smith et al., 2016, 2017b; Cho et al., 2017a; Passamonti et al., 2017; Schonhaut et al., 2017; Spina et al., 2017). However, ^18^F-AV1451 retention was also observed in non-tauopathies such as svPPA and MSA (Cho et al., 2017b; Makaretz et al., 2017). This is similar to the findings in studies of ^18^F-THK5351 PET. Off-target binding of ^18^F-AV1451 is observed in the basal ganglia, choroid plexus, and midbrain. Previous studies suggested that ^18^F-AV1451 binds with high affinity to MAO-A and neuromelanin (Marquie et al., 2015; Vermeiren et al., 2015), although this fails to account for off-target retention of this tracer in the basal ganglia. A recent study demonstrated a lack of correlation between *in vivo* ^18^F-AV1451 retention and tau pathology in cases with non-AD tauopathy (Josephs et al., 2016; Marquie et al., 2017; Smith et al., 2017a). While ^18^F-AV1451 retention was elevated in the basal ganglia of PSP cases, *in vitro* assays failed to detect significant ^18^F-AV1451 binding to 4R tau deposits in the basal ganglia of postmortem brains. It is speculated that these discrepancies are caused by the fixation of brain samples or the use of ethanol during differentiation, which can mask off-target and non-specific binding of radiotracer. Indeed, a recent autoradiography study successfully detected displaceable binding of ^18^F-AV1451 in the basal ganglia without using ethanol (Walji et al., 2016). *In vitro* binding assays demonstrated that a MAO-A inhibitor blocked ^18^F-AV1451 binding in the basal ganglia. However, the effect was relatively modest compared to that of self-blocking, indicating the presence of other off-target substrates of AV1451. ^18^F-AV1451 PET in PD patients revealed no significant differences in tracer uptake between patients who received MAO-B inhibitors (selegiline and rasagiline) and those who did not (Hansen et al., 2017), indicating that MAO-B is not an off-target substrate of ^18^F-AV1451 in the basal ganglia. Another frequent site of ^18^F-AV1451 binding is the choroid plexus which is closely located to the hippocampus. Although tau protein deposits in epithelial cells may contribute to ^18^F-AV1451 binding, it is still unclear why this tracer accumulates in the choroid plexus.

^11^C-PBB3 was developed to visualize a broad range of tauopathies in the human brain. In clinical PET studies, significant ^11^C-PBB3 retention has been detected in the hippocampus and neocortex of AD patients. ^11^C-PBB3 retention was also observed in the striatum and midbrain of PSP patients, suggesting high binding affinity of this tracer to 4R tau deposits. Off-target binding of ^11^C-PBB3 has been observed in the basal ganglia, the longitudinal sinus and the choroid plexus. The ^18^F-labeled derivatives of PBB3 (^18^F-AM-PBB3 and ^18^F-PM-PBB3) have been newly developed and showed less off-target signals in the basal ganglia and higher specific signals than ^11^C-PBB3. Recently, new-generation tau PET tracers have been developed to overcome the drawbacks of the first-generation tracers (Villemagne, 2017). ^18^F-MK6240, ^18^F-RO69558948, and ^18^F-PI2620 (**Figure 2**) also showed excellent pharmacokinetics and high binding affinity selectivity for tau aggregates in AD brain tissue (Hostetler et al., 2016; Honer et al., 2018). Preclinical binding analysis also has suggested lower binding affinity of these second-generation tracers to MAO-A and MAO-B, than the binding affinity of the first-generation tracers such as ^18^F-AV1451 and ^18^F-THK5351. A recent first-in-human study showed no detectable signal in the basal ganglia and brainstem, suggesting high binding selectivity of these tracers to tau deposition in patients with AD (Villemagne, 2017; Lohith et al., 2018; Wong et al., 2018). Further validation studies are required to confirm the binding selectivity of these tracers.

## Challenges for Imaging Other Proteinopathies

It is challenging to develop novel PET tracers for imaging other misfolded proteins such as α-synuclein and TDP-43, due to the density of binding sites of α-synuclein and TDP-43 aggregates in diseased brains is lower than that of Aβ and tau in AD brains. Like tau pathology, α-synuclein shows distinct cellular localization in synucleinopathies. Lewy bodies are observed in neurons in PD and dementia with Lewy bodies (DLB), while glial cytoplasmic inclusions (GCIs) are observed in oligodendrocytes of MSA (Shah et al., 2014). BF227 was originally developed for imaging Aβ in the brain (Kudo et al., 2007). However, this compound shows high binding affinity for recombinant α-synuclein fibrils. A ^11^C-BF227 PET study demonstrated a significant increase in tracer retention in brain regions susceptible to α-synuclein deposition in MSA (Kikuchi et al., 2010). Recently, ^11^C-PBB3 was also reported to have binding affinity to GCIs in a subset of MSA cases (Koga et al., 2017). ^11^C-PBB3 PET demonstrated significant tracer retention in MSA patients compared to age-matched control subjects (Perez-Soriano et al., 2017). Currently, no PET tracer is available for imaging Lewy bodies in the human brain. High binding affinity and selectivity for Lewy bodies are required to image Lewy pathology *in vivo*, because α-synuclein pathology is frequently concomitant with AD pathology.

Several groups have reported novel scaffold ligands that demonstrate affinity for α-synuclein aggregates. A phenothiazine derivative, ^125^I-SIL23, showed binding affinity for *in vitro* generated α-synuclein as well as PD brain homogenates (Bagchi et al., 2013). ^11^C- and ^18^F-analogs of phenothiazine derivatives appear to demonstrate insufficient binding affinity for α-synuclein in PD brain tissues. ^18^F-46a, which is a series of 3-(benzylidene)indolin-2-one, showed better selectivity for *in vitro* generated α-synuclein fibrils compared to Aβ and tau (Chu et al., 2015).

TDP-43 is frequently observed in some variants of FTLD and ALS as a primary histopathological feature. TDP-43 pathology is also concomitant with AD, PD-related disorders, Huntington’s disease, and rare disorders such as Guam ALS, as a secondary histopathological feature (Chen-Plotkin et al., 2010). TDP-43 pathology is more complicated than other proteinopathies, because there are many histopathologic subtypes, including FTLD-TDP types 1-4 and ALS-TDP (Mackenzie et al., 2010). One concern in the development of TDP-43 tracers is the density of cross β-sheet structures in TDP-43 aggregates. Although partial peptides from TDP-43 can form amyloid structures (Shimonaka et al., 2016), most TDP-43 aggregates may not have cross β-sheet structures (Cairns et al., 2007). One study reported that thioflavin-S-positive skein-like inclusions are present in only 28% of ALS cases (Robinson et al., 2013), while another study found that thioflavin-S-positive TDP-43 inclusions were present in most FTLD-TDP and ALS cases (Bigio et al., 2013). Further validation studies are required to better understand the structure of TDP-43 aggregates. If the majority of TDP-43 aggregates do not contain cross β-sheet structures, other approaches will be required (i.e., non-β-sheet ligands). Bigio et al. (2013) also observed a high density of thioflavin-S-positive astrocytosis in the superficial frontal cortex of FTLD-TDP type A. Although the significance of thioflavin-S-positive astrocytosis remains unknown, proteins containing cross β-sheet conformation might be critical in understanding the histopathology of FTLD-TDP type A.

## Conclusion

Here, we describe our experiences and lessons learned from the development of tau PET tracers, and discuss issues surrounding the development of new tracers for other proteinopathies. Advances in structural biology will aid in the development of novel PET tracers for imaging proteinopathies.

## Author Contributions

RH and NO wrote the manuscript. SF and KY revised the manuscript.

## Conflict of Interest Statement

NO owns stock of CLINO Co. Ltd. NO and SF are scientific consultants for the CLINO Co. Ltd. RH and KY have no conflict of interest.
