# Corrigendum: Imaging Protein Misfolding in the Brain Using β-Sheet Ligands

**DOI:** 10.3389/fnins.2018.00675

**Published:** 2018-09-25

**Authors:** Ryuichi Harada, Nobuyuki Okamura, Shozo Furumoto, Kazuhiko Yanai

**Affiliations:** ^1^Department of Pharmacology, Tohoku University Graduate School of Medicine, Sendai, Japan; ^2^Division of Pharmacology, Tohoku Medical and Pharmaceutical University, Sendai, Japan; ^3^Cyclotron and Radioisotope Center, Tohoku University, Sendai, Japan

**Keywords:** proteinopathies, protein aggregates, β-sheet ligands, PET, tau

In the original article, there was a mistake in Figure 2 as published. The chemical structure of [^11^C]PBB3 at top right was incorrectly described. The corrected Figure 2 appears below. The authors apologize for this error and state that this does not change the scientific conclusions of the article in any way.

**Figure 2:**
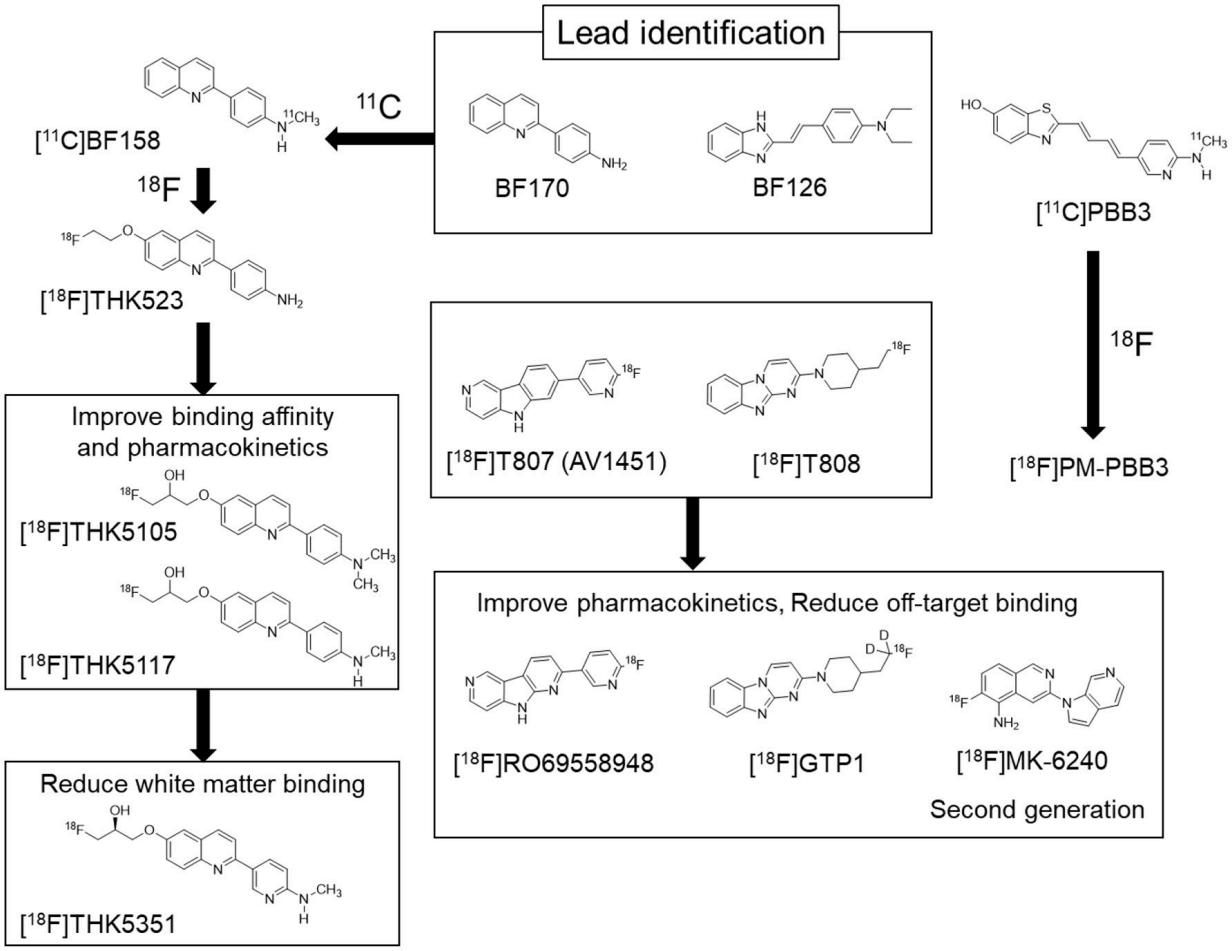
Chemical structures and flowchart of the development of tau PET tracers.

The original article has been updated.

## Conflict of Interest Statement

NO owns stock of CLINO Co. Ltd. NO and SF are scientific consultants for the CLINO Co. Ltd. RH and KY have no conflict of interest.

